# Neighbourhood out-of-home food environment, menu healthiness, and their associations with meal purchasing and diet quality: a multiverse analysis

**DOI:** 10.1186/s12937-025-01119-3

**Published:** 2025-04-10

**Authors:** Yuru Huang, Thomas Burgoine, Christine M. White, Matthew Keeble, Tom R. P. Bishop, David Hammond, Jean Adams

**Affiliations:** 1 MRC Epidemiology Unit, University of Cambridge School of Clinical Medicine, Box 285 Institute of Metabolic Science, Cambridge Biomedical Campus, Cambridge, UK; 2 Department of Human Ecology, University of California, Davis, CA USA; 3 School of Public Health Sciences, University of Waterloo, Waterloo, ON Canada; 4 Department of Marketing, Faculty of Business and Economics, University of Antwerp, Antwerp, Belgium

**Keywords:** Out-of-home, Food environment, Dietary behaviour, Multiverse analysis

## Abstract

**Background:**

Governments worldwide have implemented various interventions to improve the healthiness of food offered by out-of-home outlets. However, there is limited evidence on whether healthier menus would influence individual dietary behaviours and quality. In this cross-sectional study, we investigated associations between different measures of the neighbourhood out-of-home food environment, incorporating menu healthiness, and out-of-home meal purchasing and diet quality.

**Methods:**

We used a sample of 3,481 adults in Great Britain (GB) with valid home postcodes from the 2021 International Food Policy Study. We linked this sample to a national database of food outlet geographical locations to characterise individuals’ exposure to the out-of-home food environment. The exposure metrics included menu healthiness scores, availability, proximity, and relative composition of out-of-home food outlets in various neighbourhood buffers around the home (i.e., 500 - 1600 m). Outcomes considered were out-of-home meal consumption and overall diet quality. Using multiverse analyses, where multiple reasonable analytical choices can be tested, we investigated the associations between different exposure measures and these outcomes.

**Results:**

GB adults had access to an average of 97 (95% CI 91, 104) out-of-home food outlets within 1600 m of their homes. The number of both healthier and less healthy out-of-home food outlets was positively associated with the number of meals purchased out-of-home across all neighbourhood buffers, e.g., every 10 additional less healthy out-of-home food outlets within 500 m of the home corresponded to a 6% (95% CI = 2, 11) increase in the frequency of out-of-home meal purchases in the previous week. Proximity, relative composition, and menu healthiness of neighbourhood out-of-home outlets were not associated with out-of-home meal purchase frequency after adjusting for multiple comparisons. There were no consistent associations between out-of-home food environment exposures and diet quality.

**Conclusion:**

The only aspect of the neighbourhood out-of-home food environment associated with out-of-home meal purchase frequency was the number of out-of-home food outlets. Menu healthiness of out-of-home food outlets was not associated with how often people purchased out-of-home meals or overall diet quality. Interventions focusing on mitigating the proliferation of out-of-home food outlets may be more effective in changing individual dietary behaviour than those focusing on food served.

**Supplementary Information:**

The online version contains supplementary material available at 10.1186/s12937-025-01119-3.

## Background

Consuming food prepared outside the home is increasingly popular. In the United Kingdom (UK), individuals ate out on average 1.5 times a week, spending 31.8% of their food expenditure on consumption outside the home in 2019/2020 [1, 2]. Similarly, 55% of food expenditures in the United States (US) in 2021 and 30% in Canada in 2018 went towards out-of-home foods [3, 4]. However, foods served by out-of-home food outlets (e.g., cafés, takeaways, family/sit-down restaurants) tend to be less healthy than home-cooked meals [5]. Studies have found that out-of-home foods are high in energy, saturated fat, sugar, salt, and low in micronutrients [6–9]. Frequent out-of-home food consumption is associated with increased energy intake and higher BMI [10–13]. One potentially important factor influencing out-of-home food consumption is the food environment, including types and locations of out-of-home food outlets, and the healthiness of their offerings [14]. As such, policymakers have started to view changing the out-of-home food environment as a potential mechanism to improve population diet quality, which could contribute to lower obesity rates and better health outcomes [15, 16].

Research aimed at understanding the impact of the neighbourhood out-of-home food environment on diet and health, however, has produced mixed results in the UK and elsewhere [17–19]. Evidence remains inconclusive with respect to the association between local food environments and proximal dietary outcomes such as food consumption and purchasing [12, 20–23]. Similarly, systematic reviews have found that associations between food outlet availability and obesity are predominantly null [17, 18]. These mixed results might be explained by different aspects of the food environment being investigated, as well as the varied methodologies used to measure them.

First, previous studies investigating influences of the neighbourhood food environment predominantly focus on the geography of the food environment (i.e., whether and where an outlet exists in a neighbourhood), as opposed to accounting for what is being sold within the outlet [17, 24]. This spatial approach tends to treat broad categories of out-of-home food outlets the same; for example, while a sushi restaurant may provide healthier options compared to a pizza takeaway, this distinction between out-of-home food outlets is often lost. Furthermore, the healthiness of offerings within the same type of out-of-home food outlets may also vary.

Incorporating menu healthiness of out-of-home food outlets into measures of food environment exposure has the potential to offer a more accurate characterisation of out-of-home food outlets and may bear policy significance. Governments worldwide have implemented various interventions to help improve the healthiness of food served by out-of-home food outlets [25, 26]. Most of these interventions involve offering awards to out-of-home food outlets with healthier menus, with the intention of increasing healthy food choices on menus [25, 26]. The success of these interventions relies, in part, on the assumption that healthier menus will positively influence individual dietary behaviour, which in turn will improve individual health outcomes. However, the evidence supporting this assumption is limited, with findings largely drawn from specific settings (e.g., work cafeterias) or populations (e.g., children) in small geographical areas [27–29].

Second, mixed results in reported associations between the food environment and diet could relate to how exposure is measured [30]. Previous studies have used, for example, absolute availability (e.g., total number of food outlets), relative percentage (e.g., proportion of fast-food outlets among all out-of-home food outlets), and proximity (e.g., distance to the closest food outlet from a residential postcode) as food environment exposure measurements [30–32]. The direction, magnitude, and significance of associations between food environment exposure, diet, and weight status differ when employing different exposure metrics [30]. Additionally, studies measuring the out-of-home food environment have used a range of neighbourhood buffer specifications [33]. These include street network buffers, circular buffers, and administrative areas of different sizes [34]. The use of diverse analytical scales and exposure metrics across studies poses a challenge for synthesising and interpreting evidence. Reporting multiple measures of food environment exposure in the same study may help.

Multiverse analysis is an approach to statistical reporting that allows researchers to test and report multiple reasonable analytical choices [35, 36]. This approach is relevant to food environment research where there is no “gold standard” for what and how to measure exposure [30, 31, 37]. In this study, we used a multiverse approach to characterise multiple measures of exposure to out-of-home food outlets, incorporating menu healthiness. Our primary aim was to investigate whether healthier out-of-home food environments would influence individual dietary behaviour and quality.

## Methods

We used survey data from the International Food Policy Study (IFPS) for 3,481 participants with valid postcodes and covariates in Great Britain (GB). The survey data were then linked with Ordnance Survey (OS) Points of Interest (POI) data to characterise multiple measures of the out-of-home food environment. We conducted analyses using a multiverse approach; the detailed specifications of exposure measures, outcomes, and analytical approaches can be found in Table 1.

**Table 1:** Multiverse model dimensions

Dimension	Sub-dimension	Specification	Number of specifications
**Exposure** to neighbourhood out-of-home food outlets	Neighbourhood circular buffer size (m)	(1) 500 m (2) 1000 m (3) 1500 m (4) 1600 m (~ 1 mile)	4
	Metrics and measures	**Menu Healthiness** (1) Mean menu healthiness (2) Mean menu healthiness, weighted by distance (3) Median menu healthiness **Availability – “absolute measures”** (4) Total number of out-of-home food outlets (5) Total number of healthier^a^ out-of-home food outlets (6) Total number of less healthy^a^ out-of-home food outlets **Accessibility – “proximity measures”** (7) Distance to the closet out-of-home food outlet (8) Distance to the closet healthier^a^ out-of-home food outlet (9) Distance to the closet less healthy^a^ out-of-home food outlet **Composition – “relative measures”** (10) Proportion of healthier^a^ out-of-home food outlets (11) Proportion of less healthy^a^ out-of-home food outlets (12) Relative ratio of less healthy/healthier out-of-home food outlets	12
**Outcomes**	Out-of-home meal consumption	(1) Frequency of meals purchased away from home (2) Percentage of food prepared outside the home	2
	Overall diet quality	(1) Self-perceived overall diet healthiness (2) Healthy Diet Indicator (HDI) 2020 calculated from the 24-h dietary recall – diet survey	2
**Analytical approach**	Confounders	(1) Age, sex, ethnicity, education, perceived income adequacy, Indices of multiple deprivation (IMD) quartiles, urban/rural, and regions (2) Age, sex, ethnicity, education, perceived income adequacy, IMD quartiles, urban/rural, regions, student status, alcohol consumption, and smoking status	2
	Statistical methods	**- Frequency of meals purchased away from home last week**: negative binomial model with survey weights, modelled as count data **- Percentage of food prepared outside the home**: fraction regression model with survey weights, modelled as percentages between 0 and 1 **- Self-perceived overall diet healthiness**: ordinal logistic regression model with survey weights, modelled as ordinal data **- HDI**: generalised linear regression model with survey weights, modelled as a continuous variable	1
	Adjusting for multiple comparisons	(1) Multiple comparisons not accounted for (2) Accounted for the false discovery rate using Benjamini-Hochberg (BH) procedure	2

^a^Out-of-home food outlets in the highest tertile of menu healthiness scores were deemed healthier, and those in the lowest tertile were deemed less healthy

### Data source

#### International food policy study

Survey data was obtained from the IFPS; an annual, cross-sectional survey conducted in Australia, Canada, Mexico, the UK and United States. For our analysis, we used UK adult data from 2021. Individuals aged 18 to 100 years were eligible for the survey. Details of the data collection methods for the IFPS have been described previously [38]. Briefly, participants were recruited through Nielsen Consumer Insights Global Panel and their partner panels. Email invitations with unique survey access links were sent to a random sample of panellists after targeting for demographics; panellists known to be ineligible were not invited. Potential participants were screened for eligibility and quota requirements based on age and sex. Eligible UK participants completed web-based surveys in November – December 2021 (*N* = 4,196).

The IFPS survey comprises two components. The first is the main survey, which captures sociodemographic information, food related attitudes and behaviours. The second component is a 24-h dietary recall (hereafter referred to as the “diet survey”). The diet survey data were collected and analysed using Intake24, an automated online platform (https://intake24.org) that allows self-completion of a 24-h diet recall [39]. Participants report the time, type, and portion sizes of food and beverages consumed within a 24-h period [39]. Foods recorded in Intake24 are then linked to the UK Nutrient Composition Databank, which provides data on macronutrients, micronutrients, and food groups [41]. The IFPS received ethics clearance through a University of Waterloo Research Ethics Board (REB# 30829).

### Ordnance survey point of interest data

We obtained OS POI data containing the names and locations of out-of-home food outlets in March 2021. POI data are sourced and quality checked more than 100 data suppliers [42], however their coverage are limited to Great Britain (the UK excluding Northern Ireland). We included all out-of-home food outlets under the heading “eating and drinking”, except for “banqueting and function rooms” and “internet cafes”. These outlets included fast food and takeaway food outlets, restaurants, cafes, snack bars, tea rooms, and pubs and inns.

### Exposure measures

#### Metrics and buffers of the out-of-home food environment.

To measure a participant’s residential out-of-home food environment, we first geocoded their home address by matching their postcode to the August 2021 UK National Statistics Postcode Lookup (NSPL) [43]. The NSPL contains all postcodes in the UK and their coordinates. We then calculated multiple exposure metrics using OS POI data.

As shown in Table 1, we calculated multiple metrics of varying buffer sizes where appropriate. Specifically, we calculated absolute (count) and relative (proportion) measures, alongside accessibility (proximity), based on their precedent for use in published literature. Given the lack of consensus on a definition of residential neighbourhood scale, we also tested different Euclidean buffer sizes for absolute and relative measures [17]. A pilot study in England demonstrated that the walking neighbourhood, as perceived by participants, was often smaller than the frequently used 1 mile buffer [44]. As such, we replicated four commonly used buffer sizes: 500 m, 1000 m, 1500 m, and 1600 m (~ 1 mile).

#### Menu healthiness scores and other exposure measures

A unique aspect of this study is that we incorporated menu healthiness into our measures of the neighbourhood food environment. The menu healthiness score for each out-of-home food outlet was determined using a previously developed algorithm that predicts the healthiness score based on business names, as this is one of the few attributes available in OS POI data. Details of the algorithm have been described elsewhere [45]. Briefly, we calculated the menu healthiness scores of food outlets on *JustEat* (the leading online food delivery platform in the UK [46]) based on menu attributes. These attributes included the variety of vegetables sold, the number of fried potato (chip) mentions, and presence of special offers, desserts, salads, milk, and water [47]. We then used these as training data (*N* = 56,902 menus of unique food outlets) to develop a deep learning algorithm that predicts menu healthiness from business names. Our model achieved a mean absolute error of 0.82 on a test set (*N* = 2,729). We applied this algorithm to all out-of-home food outlets in OS POI data. Menu healthiness scores ranged from 0 to 12, with 12 being the healthiest. For example, Tom’s Fish & Chip Shop has a healthiness score of 5.39, while Yo! Sushi has a healthiness score of 9.12.

Out-of-home food outlets in the highest tertile of menu healthiness scores were deemed healthier, whereas those in the lowest tertile were deemed less healthy. This categorisation of out-of-home food outlets was incorporated into the measures of other exposure metrics (e.g., availability, accessibility, and composition), as shown in Table 1.

Combining our 12 exposure measures over four buffer sizes resulted in 39 sets of neighbourhood exposure measurements. The three distance measures (i.e., distance to the closest a) out-of-home food outlet overall, b) healthier outlet, and c) less healthy outlet) were independent of buffer size.

### Outcome measures

There were two outcomes: consumption of out-of-home food and overall diet quality.

#### Out-of-home meal consumption

We used two measures to assess out-of-home meal consumption: the *frequency* of out-of-home meal purchasing, and the *percentage* of food consumed that was prepared outside of the home. Out-of-home meal purchase frequency was assessed using the question: “During the past 7 days, how many meals did you get that were prepared away from home in places such as restaurants, fast food or take-away places, food stands, or from vending machines? Only include snacks if they counted as your meal.” Numeric responses between 0 and 21 were recorded, and responses of “Don’t know” or “Refuse to answer” were set to missing. We assessed the percentage of out-of-home food consumption from the question “Thinking about all the food you ate during the past 7 days, including snacks, what percentage was prepared outside the home?” Participants were asked to enter a percentage ranging from 0 to 100.

#### Diet quality

We used two measures to assess overall diet quality, including the self-assessed diet from the main survey as well as a diet quality indicator from the diet survey. Participants were asked to self-report “In general, how healthy is your overall diet?” with response options as follows: “Poor”, “Fair”, “Good”, “Very good”, and “Excellent”. In addition, diet quality was assessed using Intake24 (diet survey). It is estimated that participants underreport their energy intake by 22–25% when using Intake24 [48]. To mitigate underreporting, we excluded participants who completed the diet survey in less than 4 min. Previous research using Intake24, such as the National Diet and Nutrition Survey, estimated a median completion time of 12 min [40].

We used the updated Healthy Diet Indicator (HDI) to estimate diet quality, which was based on current World Health Organization (WHO) recommendations [49]. The HDI comprises 11 dietary components, including food groups and nutrients to limit. The scoring criteria for each element were either 0 or 1, and the final score ranged from 0 (least healthy) to 11 (most healthy), as presented in Table 2.

**Table 2:** Healthy diet indicator 2020 scoring criteria

Dietary element	Scoring criteria^a^
Fruits and vegetables	≥ 400 g
Beans and other legumes	> 0 g
Nuts and seeds	> 0 g
Whole grains	> 25 g
Dietary fibre	< 30% total energy
Total fat	< 10% total energy
Saturated fat	< 2 g sodium
Dietary sodium	< 10% total energy
Free sugars	< 10% total energy
Processed meat	0 g
Unprocessed meat	≤ 71 g

^a^For each of the 11 dietary elements, individuals were awarded a score of 1 for fulfilling the element and 0 for not meeting it

### Potential confounding variables

In all of our analyses, we adjusted for individual-level potential confounding variables: age, sex, ethnicity (classified as either “majority” (white alone) or “minority” (all other responses)), education level (categorised as"low"(high school completion or lower),"medium"(some post-high school qualifications), and "high"(university degree or higher)), and perceived income adequacy. Income adequacy was assessed as a categorical variable based on participants' responses to the question"Thinking about your total monthly income, how difficult or easy is it for you to make ends meet?” The possible choices ranged from “Very difficult”, “Difficult”, “Neither easy nor difficult”, “Easy”, to “Very easy”.

We also adjusted for area-level potential confounding by: deprivation, GB region (e.g., “London”, “North East”, “Wales”), and 2011 rural–urban classification [50]. Area deprivation in England was measured by the English Indices of Multiple Deprivation (IMD) at the lower super output area level [51]. Similar indices exist in Scotland and Wales [52, 53]. However, we modelled area deprivation as country-specific quartiles as indices are not directly comparable. Postcodes were coded as ‘urban’ if they were allocated to an output area with a population of 10,000 or more, or else they were classified as'rural' [50]. These area level confounders are referred to as *confounder variable set 1*.

It is possible that student status, smoking status (“no”, “yes, occasionally”, “yes, every day” in the past 30 days), and alcohol consumption frequency (“never” to “everyday”) are confounders. We additionally adjusted for these variables in *confounder variable set 2*. For all potential confounding variables, we treated responses ‘don’t know’ and ‘refuse to answer’ as missing (< 5% in total).

### Study population and statistical analysis

We only included participants with a valid home postcode in Great Britain (*N* = 3,523). Participants in Northern Ireland were excluded due to the lack of out-of-home food outlet data. Our final sample also excluded participants with missing covariates (*N* = 3,481, complete case analysis, ~ 1.2% excluded due to missingness). Among these, 2,144 participants completed the diet survey. In Supplementary File 1, we compared characteristics of the full recruited sample and the full analysed sample.

Data were weighted with post-stratification sample weights constructed using a raking algorithm, with population estimates from the census based on age group, sex, region, ethnicity, and education. We used the same set of survey weights for the overall sample and the subsample that completed the diet survey since the distribution of the weighting variables was similar. Survey weights were rescaled to sample size for descriptive statistics. Estimates reported were weighted unless otherwise specified.

Statistical models were selected based on the outcome being investigated. To explore the relationship between exposure to the out-of-home food environment and the frequency of meals purchased away from home, we used negative binomial models, given that meal purchasing frequency was overdispersed count data. Given that the percentage of food prepared outside the home was a response between 0 and 1, we used fraction regression models for this outcome. For self-perceived overall diet healthiness, we applied ordinal logistic regression models. Generalised linear models were used to model the relationship between exposure measurements to HDI. Although we attempted to address the potential for residual confounding by using an instrumental variable approach, we were unable to identify a valid instrument for our analyses [54] (Supplementary File 2).

We also applied a series of transformations to the exposure measurements in statistical models to improve the interpretability of our findings. These transformations included normalising the menu healthiness metrics, counting the availability metrics in multiples of 10, measuring accessibility in increments of 100 m, and expressing proportions (measure of composition) in units of 10%. The results can therefore be interpreted as the change in outcome for 1 SD increase in menu healthiness measures, 10 additional out-of-home food outlets, 100 m increase in distance variables, and 10% increase in proportions.

To minimise the false discovery rate, we applied the Benjamini-Hochberg (BH) procedure; although we also present results without adjustment [55]. The BH procedure helps decrease the number of false positives arising from multiple significance testing.

All statistical analyses were conducted in R version 4.2.2 (Vienna, Austria).

## Results

### Characteristics of study participants

Table 3 shows descriptive weighted statistics for the main analytical sample (*N* = 3,481) and diet sample (*N* = 2,144). The median age of the main analytical sample was 51 years (Interquartile range (IQR) 35, 64) and approximately half of the participants were female (50.2%). The majority of the main analytical sample were White (89.6%), had a high school degree or lower (50.4%), and reported that they had enough income to make ends meet (neither easy nor difficult or above, 79.3%). Most participants included in the main analytical sample lived in urban areas (84.7%), with the highest concentration in the South East, London and the North West regions (combined 37.8%). The sociodemographic characteristics of the diet sample were largely similar to those of the main analytical sample.

**Table 3:** Sociodemographic characteristics and outcomes in the full analytical sample and diet subsample

	**Labels**	***Main analytical sample* (*N* = 3,481)**^a^	***Diet Sample* (*N* = 2,144)**^a^
**Sociodemographic characteristics**			
** Age**^b^		51 (35, 64)	55 (40, 66)
** Sex (%)**	Male	1,735 (49.8)	1,014 (47.3)
	Female	1,746 (50.2)	1,130 (52.7)
** Ethnicity (%)**	Majority: White	3,120 (89.6)	1,934 (90.2)
	Minority: all other responses	361 (10.4)	210 (9.8)
** Education (%)**	Low: high school completion or lower	1,754 (50.4)	1,053 (49.1)
	Medium: some post-high school qualifications	733 (21.1)	468 (21.8)
	High: university degree or higher	993 (28.5)	623 (29.1)
** Income Adequacy (%)**	Very difficult	190 (5.5)	110 (5.1)
	Difficult	530 (15.2)	332 (15.5)
	Neither easy nor difficult	1,230 (35.3)	735 (34.3)
	Easy	915 (26.3)	586 (27.3)
	Very easy	616 (17.7)	382 (17.8)
** IMD Ranking (%)**	Q1: least deprived	1,003 (28.8)	580 (27.1)
	Q2	891 (25.6)	537 (25.1)
	Q3	846 (24.3)	533 (24.8)
	Q4: most deprived	742 (21.3)	494 (23.0)
** Urban/Rural (%)**	Rural	534 (15.3)	367 (17.1)
	Urban	2,947 (84.7)	1,777 (82.9)
** Regions (%)**	North East	146 (4.2)	92 (4.3)
	North West	394 (11.3)	257 (12.0)
	Yorkshire and the Humber	295 (8.5)	183 (8.5)
	East Midlands	275 (7.9)	154 (7.2)
	West Midlands	321 (9.2)	193 (9.0)
	East	335 (9.6)	215 (10.0)
	London	439 (12.6)	252 (11.8)
	South East	485 (13.9)	305 (14.2)
	South West	311 (8.9)	197 (9.2)
	Scotland	303 (8.7)	181 (8.4)
	Wales	176 (5.1)	115 (5.4)
**Outcome Variables**			
**Frequency of meals purchased away from home in the past 7 days**^b^		1 (0, 3)	1 (0.0, 2)
**Percentage of food prepared out-of-home in the past 7 days**^b^		5 (0,20)	5 (0, 20)
**Self-assessed diet quality (%)**	Poor	243 (7.1)	141 (6.6)
	Fair	977 (28.4)	654 (30.7)
	Good	1,388 (40.4)	857 (40.2)
	Very good	659 (19.2)	399 (18.7)
	Excellent	170 (4.9)	80 (3.7)
**Healthy Diet Index**^b^			4 (3, 5)

^a^The total number may vary slightly (either higher or lower) from the sample size due to rounding after weighting

^b^Median values and interquartile range were shown for these continuous variables

In terms of the outcome variables, in both the main analytical sample and the diet sample, the median number of meals purchased outside the home in the past 7 days was 1 (IQR = 0, 3 main analytical sample, IQR = 0, 2 diet sample). The median percentage of food, including snacks, that was prepared out-of-home was 5% (IQR = 0, 20). The largest proportion of participants reported their diet as “good” (40.4% in the main analytical sample and 40.2% in the diet sample), followed by “fair” and “very good”. In the diet sample where we evaluated the HDI using 24-h recall data, the median score is 4 (IQR 3, 5), on a scale that ranges from 0 to 11.

### Exposure to the out-of-home food environment

#### Exposure metrics using different neighbourhood buffer sizes

Table 4 provides a summary of all exposure measures at different buffer sizes in the main analytical sample. As the buffer size increased, menu healthiness and availability metrics also increased. The mean normalised menu healthiness score increased from − 0.13 (500 m buffer, 95% CI = − 0.19, − 0.07) to 0.01 (1600 m buffer, 95% CI = − 0.02, 0.04). Whereas participants had access to a mean of 14 (95% CI = 13, 15) outlets within 500 m of their home, at 1600 m buffer, participants had access to a mean of 97 (95% CI = 91, 104) out-of-home food outlets on average based on our definition. While the composition of out-of-home food outlets remained relatively consistent across buffer sizes, the number of less healthy out-of-home food outlets exceeded that of healthier ones across buffer sizes (e.g., 500 m buffer, Relative Ratio = 1.38, 95%CI = 1.3, 1.5). The mean distance to the nearest out-of-home food outlet was 347 m (95% CI = 330, 363). The mean distance to the closest *healthier* outlet was further away compared to the closest less healthy outlet, but the two did not differ significantly. Within a 1600 m buffer, different exposures were strongly correlated within the same dimension (e.g., healthiness) but showed weaker associations across dimensions (e.g., proximity vs. availability) (Supplementary File 3).

**Table 4:** Exposure metrics by buffer size

Buffer Size	500 m	1000 m	1500 m	1600 m
**Menu Healthiness Scores**				
Mean normalised menu healthiness scores (95% CI)	− 0.13 (− 0.19, − 0.07)	− 0.04 (− 0.08, 0.00)	0.00 (− 0.03, 0.03)	0.01 (− 0.02, 0.04)
Mean weighted normalised menu healthiness scores (95% CI)	− 0.11 (− 0.17, − 0.06)	− 0.04 (− 0.08, 0.00)	0.00 (− 0.03, 0.03)	0.00 (− 0.03, 0.03)
Median normalised menu healthiness scores (95% CI)	− 0.19 (− 0.25, − 0.12)	− 0.05 (− 0.09, − 0.01)	0.03 (0.00, 0.06)	0.04 (0.01, 0.07)
**Availability**				
Total number of OOHFO (95% CI)	14 (13, 15)	45 (42, 48)	88 (82, 94)	97 (91, 104)
Number of healthier^a^ OOHFO (95% CI)	6 (5.4, 6.7)	17 (15, 18)	32 (29, 35)	36 (32, 39)
Number of less healthy^a^ OOHFO (95% CI)	6 (5.2, 6.0)	15 (14, 16)	29 (27, 30)	32 (30, 34)
**Composition**				
Proportion of healthier^a^ OOHFO (95% CI)	0.29 (0.28, 0.30)	0.31 (0.30, 0.31)	0.31 (0.30, 0.32)	0.31 (0.30, 0.32)
Proportion of less healthy^a^ OOHFO (95% CI)	0.39 (0.37, 0.40)	0.37 (0.37, 0.38)	0.36 (0.36, 0.37)	0.36 (0.36, 0.37)
Relative ratio of less healthy/healthier out-of-home food outlets (95%CI)	1.38 (1.3, 1.5)	1.58 (1.5, 1.6)	1.47 (1.4, 1.5)	1.45 (1.4, 1.5)
**Accessibility**				
Distance to the closest OOHFO (95%CI)	347 (330, 364)			
Distance to the closest healthier^a^ OOHFO (95% CI)	596 (562, 629)			
Distance to the closest less healthy^a^ OOHFO (95% CI)	585 (548, 623)			

^a^Out-of-home food outlets (OOHFO) in the highest tertile of menu healthiness scores were deemed healthier, and those in the lowest tertile were deemed less healthy

#### (A subset of) exposure metrics by individual sociodemographic variables

**Fig. 1:**
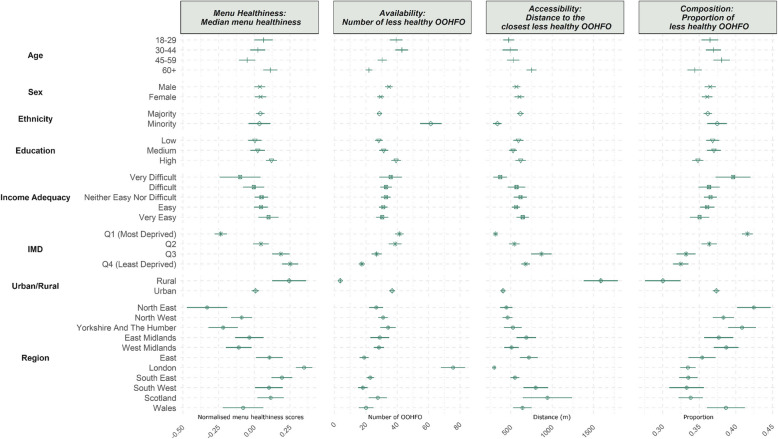
A subset of out-of-home food environment measures within 1600 m of homes based on sociodemographic characteristics

To illustrate how exposure varied by sociodemographic characteristics (*confounder set 1*), we chose one metric to represent each dimension of the out-of-home food environment within a 1600 m buffer, as shown in Fig. 1. These are descriptive statistics and not adjusted for any confounding variables.

Although the patterns varied across exposure metrics, in general, more socioeconomically disadvantaged individuals were exposed to less healthy out-of-home food environments (Fig. 1). This was most evident for area deprivation: individuals living in more deprived areas were exposed to more and higher percentages of less healthy out-of-home food outlets. Income adequacy showed similar patterns. However, not all metrics showed a clear pattern. For example, although ethnic minorities were exposed to a higher number of less healthy food outlets, the median menu healthiness scores of their neighbourhood out-of-home food outlets and the proportion of less healthy out-of-home food outlets were not statistically different from the ethnic majority (judged by overlapping CI). Despite being exposed to the highest number of less healthy food outlets, individuals with the highest level of education, or individuals residing in London also had the highest menu healthiness scores of out-of-home food outlets around home. This suggests that different exposure metrics may capture different aspects of the food environment and are not interchangeable.

### Associations between exposure to out-of-home food environment and out-of-home meal purchasing

**Fig. 2:**
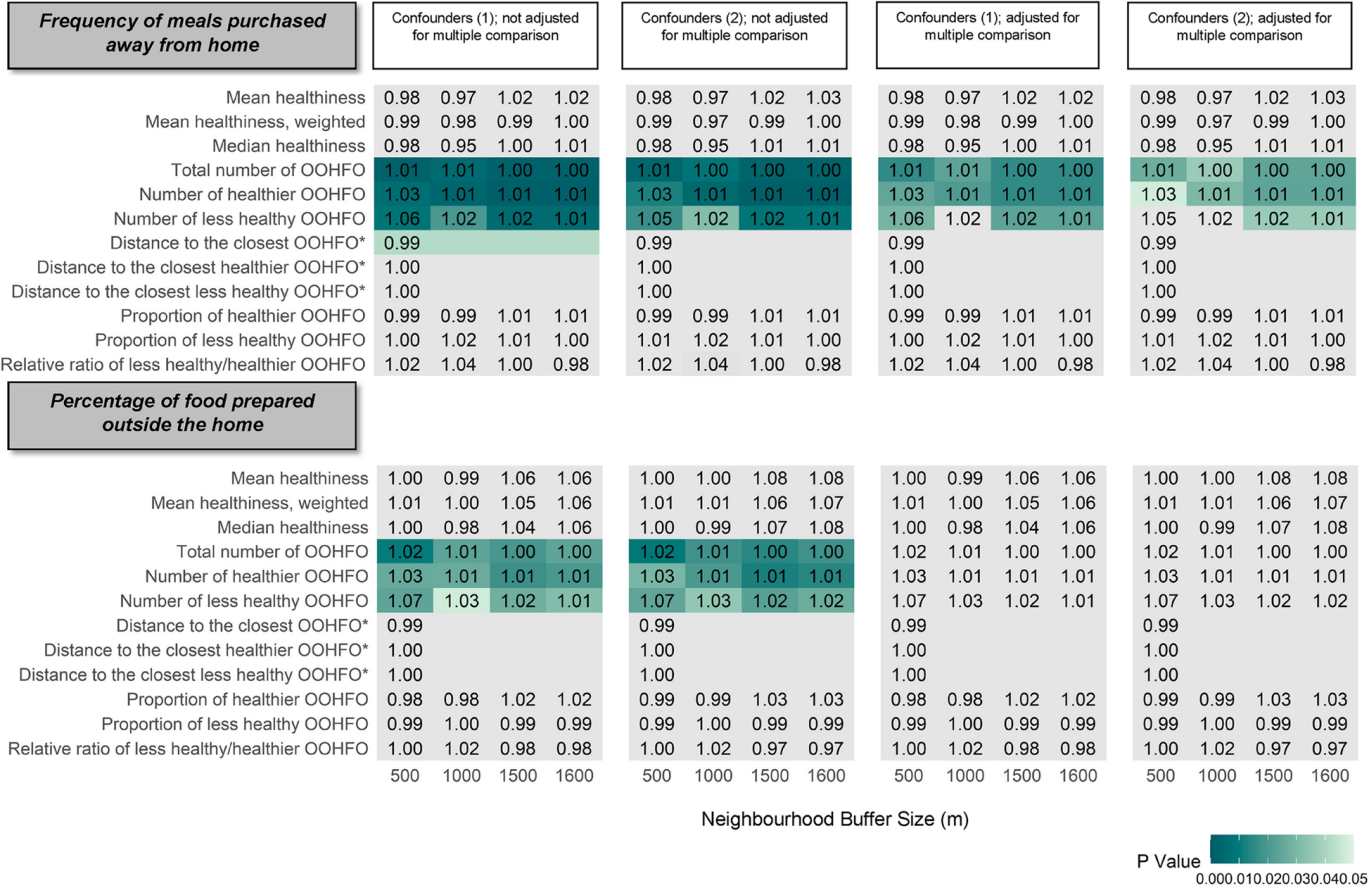
Associations between the neighbourhood out-of-home food environment and out-of-home meal consumption. Coefficients denote the relative change in the expected outcome (incidence rate ratio, IRR) for meal purchasing frequency and odds ratios for the percentage of food prepared out-of-home, with one SD increase in healthiness scores, 10 more out-of-home food outlets (OOHFO), 100 m increase of the distance variables, and 10% increase in proportions. Associations not statistically significant are in grey. Multiple comparisons were adjusted for using the Benjamini–Hochberg Procedure. Confounders (1): Age, sex, ethnicity, education, perceived income adequacy, IMD quartiles, urban/rural, regions; Confounders (2): Age, sex, ethnicity, education, perceived income adequacy, IMD quartiles, student status, alcohol consumption, smoking status, and regions. * Distance metrics are independent of buffer size

As shown in Fig. 2, the number of out-of-home food outlets, regardless of their healthiness, was positively associated with out-of-home meal purchase frequency in the past 7 days. For example, 10 additional total food outlets within 500 m were associated with a 1.47% (95%CI = 0.55, 2.40) greater frequency of out-of-home meal purchases after adjusting for confounding set 1. The percentage difference was 6.21% (95%CI = 1.57, 11.06) for 10 additional less healthy out-of-home food outlets and 2.78% (95%CI = 0.78, 4.83) for 10 additional healthier ones. The only exceptions were the number of less healthy out-of-home food outlets within 500 m and 1000 m of participants’ homes when accounting for multiple comparisons.

The percentage of food purchased out-of-home was also positively associated with the availability of total, healthier, and less healthy out-of-home food outlets without accounting for multiple comparisons. For 10 additional out-of-home food outlets within 500 m of home, the odds of expected out-of-home food percentages are multiplied by 1.07 (95%CI = 1.01, 1.14). However, these correlations were not statistically significance once multiple comparisons were taken into account.

### Associations between exposure to the out-of-home food environment and diet quality

**Fig. 3:**
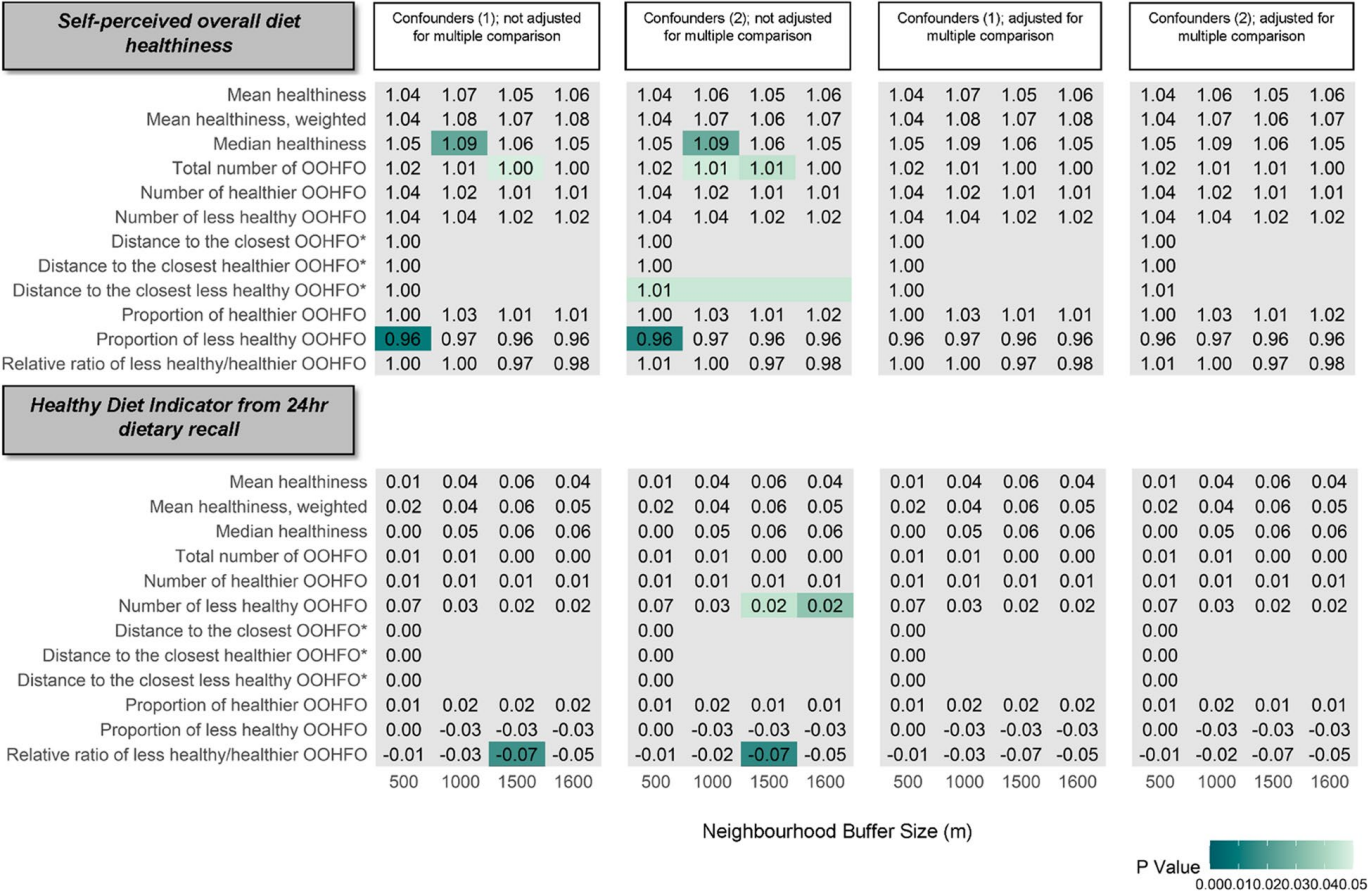
Associations between exposure to out-of-home food (OOHFO) and overall diet quality. Coefficients denote the odds of improved diets (moving up one category, for self-perceived diet), or increase in healthy diet indicator (HDI) score, with one SD increase in healthiness scores, 10 more out-of-home food outlets (OOHFO), 100 m increase in distance variables, and 10% increase in proportions. Associations not statistically significant are in grey. Multiple comparisons were adjusted for using the Benjamini–Hochberg Procedure. Confounders (1): Age, sex, ethnicity, education, perceived income adequacy, IMD quartiles, urban/rural, regions; Confounders (2): Age, sex, ethnicity, education, perceived income adequacy, IMD quartiles, student status, alcohol consumption, smoking status, and regions. * Distance metrics are independent of buffer size

The relationship between exposure to the out-of-home food environment and overall diet quality were sporadic and less consistent (Fig. 3). Results showed that when multiple comparisons were not accounted for, self-perceived diet healthiness was positively associated with median menu healthiness (OR = 1.09, 95% CI = 1.01, 1.18) at 1000 m buffer and total number of out-of-home food outlets (OR = 1.00, 95% CI = 1.00, 1.01) at 1500 m buffer, and negatively associated with proportion of less healthy food outlets (OR = 0.96, 95% CI = 0.94, 0.99) of food outlets at 500 m buffer, adjusted for confounders variable set 1.

Similarly, the HDI derived from the diet survey was associated with some menu healthiness, distance, and availability metrics, but not consistently: for example, across both confounder sets, relative ratio of less healthy/healthier out-of-home food outlet was negatively associated with HDI score (− 0.07, 95% CI = − 0.13, − 0.01, the confounders variable set 1). These associations were not statistically significant after accounting for multiple comparisons.

## Discussion

### Summary of findings

To the best of our knowledge, this is the first study to incorporate menu healthiness as a measure of the out-of-home food environment and examine its association with dietary behaviour and quality on a national scale. In addition to menu healthiness, we also calculated multiple exposure metrics including availability, accessibility, and composition, using different neighbourhood buffer sizes, which we integrated using a multiverse approach. We found that the number of food outlets in the residential neighbourhood, regardless of their healthiness, was associated with increased frequency of out-of-home meal purchasing. This was the only aspect of the out-of-home food environment that showed a consistent significant association with dietary behaviour after accounting for multiple comparisons. The relationship between exposure to the out-of-home food environment and overall diet quality was inconsistent, with some weak associations that were not statistically significant after controlling for multiple comparisons. Menu healthiness was not associated with out-of-home meal purchasing nor diet quality when multiple comparisons was taken into account.

### Strength & limitations

A major strength of this study is the incorporation of menu healthiness into measurement of out-of-home food environment. Its inclusion provides a more nuanced understanding of neighbourhood food environment exposure, compared to focusing solely on the spatial distribution of all out-of-home food outlets. Another strength of our study is that we used a multiverse approach, which allowed us to consider multiple exposure measures, outcomes, and analytical methods. Rather than reporting one set of results, we analysed and presented the results of multiple reasonable approaches to specifying both exposure and outcomes in a systematic fashion [36]. As previously mentioned, research studies investigating the influences of the out-of-home food environment on diet health have found mixed results [12, 20, 22], which could be explained by variability in exposure and outcome specifications. While the availability of data may limit what we can test, when several reasonable alternatives exist, there is no reason to exclude these from our analysis. We made every effort to narrow down the number of these alternatives based on past literature and theory. This multiverse approach facilitates side-by-side comparisons, thereby increasing the transparency of our study.

Our study, however, was not without limitations. Firstly, our assessment of menu healthiness was based on broad menu attributes and may not have captured finer details such as cooking methods, type of oil used, types of pasta (e.g., white or whole grain), or rice (e.g., brown rice or white rice), which are aspects of some healthy catering interventions [26]. More research is needed that incorporates these details to improve menu healthiness estimates. Secondly, it is possible that a threshold effect is at play in dietary behaviour change. For instance, it may require a certain threshold percentage of healthy foods on a menu to trigger a change in behaviour. However, current evidence suggests that most out-of-home items are unhealthy, which limits the possible effect [56, 57]. In addition, we categorised out-of-home food outlets into tertiles, which is a relative assessment rather than an absolute one. The top tertile in terms of healthiness might still be relatively unhealthy. However, assessing *absolute healthiness of out-of-home food outlet menu* is challenging, and there is no gold standard for it. While we aimed to comprehensively characterise exposure to out-of-home food outlets, our approach is not exhaustive. Future research could, for example, examine relative measures of access to different types of food outlets, such as the percentage of takeaways.

Our study also has some limitations regarding the food outlets included. We only considered physical food outlets listed in the Ordnance Survey, and did not capture informal food outlets (e.g., food trucks) that may influence individual dietary behaviors [58, 59]. However, due to a lack of available data, we were unable to account for these outlets. Additionally, with the growing popularity of online food delivery services, their impact on the food environment is an important consideration [60]. While this study focuses on physical food environments, future research should explore the interplay between physical and online food outlets and incorporate online measures into food environment assessments.

Additionally, relying on 24 h dietary recall may be subject to recall bias and underreporting. For example, it has been shown that participants under-report energy intake by 25% in Intake24 [48]. We attempted to mitigate this issue by excluding those who completed the recall within 4 min. Frequency of out-of-home consumption is also a crude estimate subject to recall bias. Linking neighbourhood exposure to more granular food purchasing data from out-of-home food outlets could potentially offer a more accurate assessment of this relationship. Furthermore, our study did not consider other aspects of the food environment, such as supermarket and other food retail exposure, or food outlet exposure in non-residential neighbourhoods. Given the collinearity of different metrics, we were also unable to investigate the combined associations of availability, proximity, composition, and menu healthiness. A composite food environment index incorporating these metrics, along with other dimensions of the food environment, such as the price of food sold and promotional offers, could potentially prove useful for understanding the complexities in the out-of-home food environment. Moreover, it is also possible that the true effect size of out-of-home food environment exposure on diet is modest, and may not have been detectable with our sample size. We did observe some weak associations between menu healthiness metrics and diet quality prior to accounting for multiple comparisons. Lastly, our analyses were based on cross-sectional data collected in 2021, a period when COVID- 19 may have influenced both the out-of-home food environment and dietary behaviours. Future research could determine whether these patterns persist in subsequent years and assess the long-term impact of changes in the food environment on diet quality.

### Interpretation of findings

We did not find any consistent patterns of association between menu healthiness and diet quality. In out-of-home settings, one study found limited associations between the nutrition environment of restaurants and diet quality in US children [29]. A possible explanation is that individuals may not opt for healthier food choices in more favourable (healthier) out-of-home consumer environments. Simply increasing the healthiness of menus at local out-of-home food outlets may not necessarily lead to a shift in people’s food purchases. Eating out can represent a way for people to escape their routine, with little concern over whether or not the food they select is healthy [61, 62]. In a study conducted in Australian fast food restaurants, despite the availability of healthier options on the menu, only 1% of meal purchases included any of these [63].

Another possibility is that people do make healthier choices in healthier out-of-home food outlets, but that because out-of-home food represents a relatively small part of the total diet (a median value of 5% in our sample), it may not be significantly affected. The proportion of meals purchased from out-of-home food outlets may have been lower in our study, as our data collection coincided with the COVID- 19 omicron outbreak in the UK. Although there were no mandatory restaurant closures at this time, the surge in cases and safety measures—such as mask mandates and social distancing—may have reduced the popularity of dining out, thereby lowering this percentage as well as potentially influencing individuals’ dietary behaviour when eating outside the home. Alternatively, they might adjust other eating habits to compensate for healthier choices made outside the home [64].

As highlighted in the limitations, the inconsistent findings between menu healthiness measures for food outlets and dietary outcomes may also be attributed to the methodology used to develop these scores. Since we were only able to capture broad attributes of menus—without detailed information on nutritional composition, processing methods, and other factors relevant to the healthfulness of menu items—this limitation may have led to errors and attenuation of parameter estimates. However, it is important to note that all associations between menu healthiness and dietary behaviours align with expected directions, even if they do not reach statistical significance. Specifically, greater exposure to healthier food outlets in neighbourhoods is associated with reduced out-of-home food purchasing and improved diet quality. This suggests face validity of our menu healthiness measure and that menu healthiness could play a meaningful role in dietary behaviour, perhaps despite our study having been underpowered to detect significant effects.

On the other hand, the availability of out-of-home food outlets, irrespective of healthiness of menu, was the only aspect of the food environment associated with dietary behaviour. Specifically, the number of healthier, less healthy, and total out-of-home food outlets were all positively associated with the frequency of meals purchased outside the home. This is consistent with earlier research that identified an association between greater exposure to takeaways and consumption of takeaway food [12]. It is also worth noting that the availability of less healthy out-of-home food outlets had a more pronounced association with purchase frequency than the availability of healthier ones, particularly within a 500 m radius of home (6% vs 3% increase in purchasing frequency). This suggests that less healthy out-of-home food outlets may be a greater motivator for people to purchase more out-of-home meals compared to healthier food outlets. It could be that less healthy out-of-home food outlets, such as fast-food outlets, are perceived as inexpensive, friendly, and more convenient [65, 66]. While associations generally remained consistent across buffer sizes, the effect sizes we found were larger in smaller neighbourhood buffers. In a systematic review, the authors found no association between food accessibility and food consumption beyond a 500 m buffer of household locations [33]. It is possible that smaller buffers more precisely capture the spatial extent of food purchasing behaviours, and hence have a stronger association with dietary behaviour [67, 68].

While availability of out-of-home food outlets was positively associated with frequency of out-of-home meal consumption, we did not find any consistent significant correlation between availability and diet quality after accounting for multiple comparisons. Furthermore, we observed an unexpected positive relationship between availability of less healthy food outlets and diet quality when using larger buffer sizes before adjustment for multiple comparisons. This may be attributed to residual confounding, where this weak association could be caused by other unmeasured individual or neighbourhood characteristics that are linked to both the exposure and outcome. A recent systematic review examining activity space food environment exposures using GPS data also found no consistent patterns between exposure and diet-related outcomes in the international literature [69].

Our analyses also revealed that larger buffer sizes were associated with higher menu healthiness scores, suggesting that out-of-home food outlets closer to homes may offer less healthy menu options. This might be due to healthier outlets often being more expensive and therefore having a smaller market [70]. Consequently, they tend to be concentrated in town centres, whereas less healthy ones could be more economically viable in all types of neighbourhoods.

Additionally, individuals who were more socially vulnerable, such as those with lower income adequacy, were exposed to a less healthy out-of-home food environment. This has been found in past food environment studies in the UK [71, 72]. For example, one study reported higher percentages of takeaway food outlets in their neighbourhoods among participants with lower levels of education and income [71]. This observed pattern could contribute to the double burden of low income and an unhealthy food environment, as individuals with lower income were also more susceptible to exposure to unhealthy out-of-home food outlets [72].

### Implications for research and policy

Our findings indicate that out-of-home food environment exposure metrics are not interchangeable. The choice of exposure metric and neighbourhood buffer size may lead to distinctly different outcomes and interpretations. For example, proximity, menu healthiness, and composition metrics showed little association with out-of-home food consumption frequency, while associations were consistently statistically significant for availability metrics. This finding is in accordance with a systematic review that reported how availability tended to have a greater effect on dietary behaviour than measures of proximity [31]. Researchers should be aware of how their methodological choices matter.

While many interventions have aimed to promote healthier out-of-home meals in the UK [25], our study did not find sufficient evidence to support the notion that healthier menus translate into healthier overall diet. That is not to say that menu healthiness does not matter, but rather that based on the current evidence, along with our findings, its impact on overall diet might be minimal, if present. Policies that aim to curb the proliferation of out-of-home food outlets, however, may reduce the frequency of out-of-home eating. An example of such a policy is the use of the urban planning system to restrict opening of new takeaway food outlets [73].

## Conclusion

Among the different measures used to assess menu healthiness, availability, accessibility, and composition of the neighbourhood out-of-home food environment, the only aspect associated with out-of-home meal purchasing was the number of out-of-home food outlets. Menu healthiness of out-of-home food outlets was not associated with how often adults living in GB purchased out-of-home meals or their overall diet quality in 2021. Interventions focusing on mitigating the proliferation of out-of-home food outlets may be more effective in changing individual dietary behaviour than those focusing on food served.

## Supplementary Information

Supplementary Material 1. Information about the study sample; weighted individual characteristics of the full recruited and analysed samples.

Supplementary Material 2. Instrumental variable approach; describes the authors’ attempt to apply the instrumental variable method.

Supplementary Material 3. A correlation plot showing the correlations between different exposure measures within a 1600m buffer.

## Data Availability

The datasets generated and/or analysed during the current study are available from the corresponding author upon reasonable requests.

## Abbreviations

UK: United Kingdom
IFPS: International Food Policy Study
GB: Great Britain
OS: Ordnance Survey
POI: Points of Interest
NSPL: National Statistics Postcode Lookup
HDI: Healthy Diet Indicator
WHO: World Health Organization
IMD: Indices of Multiple Deprivation
BH: Benjamini-Hochberg
IQR: Interquartile range
OOHFO: Out-of-home food outlets
