# Supplementary material for: Neighbourhood out-of-home food environment, menu healthiness, and their associations with meal purchasing and diet quality: a multiverse analysis
Source: Nutr J. 2025 Apr 10;24:56. doi: 10.1186/s12937-025-01119-3 (PMC11983832; doi:10.1186/s12937-025-01119-3)
Supplement: Supplementary file 1 — Supplementary Material 1. Information about the study sample; weighted individual characteristics of the full recruited and analysed samples. [file 12937_2025_1119_MOESM1_ESM.docx]

Information about the study sample

Detailed information about how the sample was recruited and its representativeness of the target group is available in the International Food Policy Study’s methodology for the 2021 adult surveys: https://foodpolicystudy.com/methods/.

**Table S1** presents the weighted characteristics of both the full recruited and analysed samples.

Since not all of the recruited sample has a valid postcode, we cannot determine the IMD and rural/urban of their residence. As illustrated in **Table S1**, the characteristics of the weighted analysed sample were similar to those of the recruited sample.

**Table 1 Weighted characteristics of the recruited sample and the analysed study sample**

|  | **Labels** | ***Analysed study Sample***  **(N = 3,481)** | ***Recruited Sample***  **(N = 4,196)** |
| --- | --- | --- | --- |
| **Age**  **(median, IQR)** | | 51 (35, 64) | 49 (34, 64) |
| **Sex (%)** | Male | 1,735 (49.8) | 2,052 (48.9) |
|  | Female | 1,746 (50.2) | 2,144 (51.1) |
| **Ethnicity (%)** | Majority: White | 3,120 (89.6) | 3,702 (88.2) |
|  | Minority: all other responses | 361 (10.4) | 470 (11.2) |
|  | Not stated |  | 25 (0.6) |
| **Education (%)** | Low: high school completion or lower | 1,754 (50.4) | 2,087 (49.8) |
|  | Medium: some post-high school qualifications | 733 (21.1) | 888 (21.2) |
|  | High: university degree or higher | 993 (28.5) | 1,182 (28.2) |
|  | Not stated |  | 37 (0.9) |
| **Income Adequacy (%)** | Very difficult | 190 (5.5) | 248 (5.9) |
|  | Difficult | 530 (15.2) | 650 (15.5) |
|  | Neither easy nor difficult | 1,230 (35.3) | 1,494 (35.6) |
|  | Easy | 915 (26.3) | 1,051 (25.0) |
|  | Very easy | 616 (17.7) | 699 (16.6) |
|  | Missing |  | 54 (1.3) |
| **Regions (%)** | North East | 146 (4.2) | 170 (4.1) |
|  | North West | 394 (11.3) | 460 (11.0) |
|  | Yorkshire and the Humber | 295 (8.5) | 345 (8.2) |
|  | East Midlands | 275 (7.9) | 306 (7.3) |
|  | West Midlands | 321 (9.2) | 369 (8.8) |
|  | East | 335 (9.6) | 390 (9.3) |
|  | London | 439 (12.6) | 552 (13.1) |
|  | South East | 485 (13.9) | 574 (13.7) |
|  | South West | 311 (8.9) | 361 (8.6) |
|  | Scotland | 303 (8.7) | 352 (8.4) |
|  | Wales | 176 (5.1) | 201 (4.8) |
|  | Northern Ireland |  | 115 (2.7) |
