# Supplementary material for: Neighbourhood out-of-home food environment, menu healthiness, and their associations with meal purchasing and diet quality: a multiverse analysis
Source: Nutr J. 2025 Apr 10;24:56. doi: 10.1186/s12937-025-01119-3 (PMC11983832; doi:10.1186/s12937-025-01119-3)
Supplement: Supplementary file 2 — Supplementary Material 2. Instrumental variable approach; describes the authors’ attempt to apply the instrumental variable method. [file 12937_2025_1119_MOESM2_ESM.docx]

Instrumental variable approach

# **Instrumental Variables**

The relationship between exposure to out-of-home food outlets and dietary behaviour may be confounded by unobserved confounders, in addition to observed biases.^1^ Instrumental variable (IV) regression is one of the causal methods used to address such unobserved biases, or, residual confounding. The main idea behind this approach is to identify an instrumental variable that is associated with the exposure but not the outcome, hence randomising the assignment (“exposure”). We hypothesised that a participant’s *school density* and *neighbourhood walkability* could be used as instruments, with the definition of “neighbourhood” consistent with how exposure was defined. In other words, we hypothesised these two variables were associated with our measured exposure to the out-of-home food environment, and not directly associated with the outcomes. School density was calculated for each participant and buffer size using OS POI data. Neighbourhood walkability scores were obtained from Walk Score using their free Application Programming Interface (API).^2^

# **Validity of Instrumental Variables**

To determine the validity of our selected instruments, we ran the weak instrument test (i.e., F-statistic) and Wu–Hausman test for endogeneity. A good instrument should be a strong instrument and not associated with the residuals. We found that both school density and walkability score were weak instruments, as they were not strongly associated with either out-of-home food consumption or overall diet quality, except for few instances (e.g., total number of schools was not a weak instrument for mean weighted normalised menu healthiness score at 500m buffer and eating out frequency, however Wu-Hausman test suggests that OS model could be used in this case). As such, we were unable to find a valid instrument in our study across different exposure measures, therefore did not use an IV approach for our analyses.

# **References**

1. Rummo PE, Guilkey DK, Ng SW, et al. Understanding bias in relationships between the food environment and diet quality: the Coronary Artery Risk Development in Young Adults (CARDIA) study. *J Epidemiol Community Health* 2017;71(12):1185-90. doi: 10.1136/jech-2017-209158 [published Online First: 2017/10/07]

2. Walk Score [Available from: <https://www.walkscore.com/> accessed Nov 14 2022.
