# Supplementary material for: Neighbourhood out-of-home food environment, menu healthiness, and their associations with meal purchasing and diet quality: a multiverse analysis
Source: Nutr J. 2025 Apr 10;24:56. doi: 10.1186/s12937-025-01119-3 (PMC11983832; doi:10.1186/s12937-025-01119-3)
Supplement: Supplementary file 3 — Supplementary Material 3. A correlation plot showing the correlations between different exposure measures within a 1600m buffer. [file 12937_2025_1119_MOESM3_ESM.docx]

Correlations among exposure measures

The correlation plot below shows the correlations between different exposure measures within a 1600m buffer. Within a 1600m buffer, different exposures were strongly correlated within the same dimension (e.g., healthiness) but showed weaker associations across dimensions (e.g., proximity vs. availability).


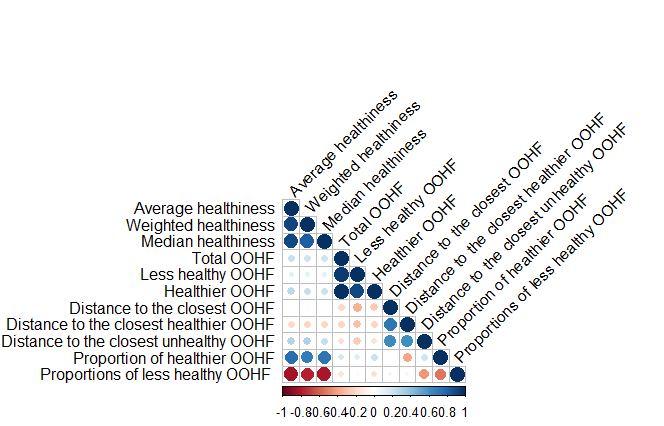


**Figure e1** Correlation plot for exposure measures at 1600m buffer. *Abbreviation*: out-of-home food outlets (OOHF)
